# LIVE DISCHARGE FROM HOSPICE WITH DEMENTIA: CHALLENGES TO SUPPORTING THE PATIENT-CAREGIVER DYAD

**DOI:** 10.1093/geroni/igac059.2514

**Published:** 2022-12-20

**Authors:** Stephanie P Wladkowski, Cara L Wallace, Karla Washington

**Affiliations:** Bowling Green State University, Bowling Green, Ohio, United States; Saint Louis University, Saint Louis, Missouri, United States; Washington University in St. Louis, St. Louis, Missouri, United States

## Abstract

A live discharge from hospice disrupts care continuity and results in burdensome transitions for individuals with a life-limiting illness and their caregivers. In 2019, hospices served more than 1.6 million people across the United States, with nearly 63% of Medicare decedents age 85 or older. Of these patients, nearly 350,000 (20.9%) had a principal diagnosis of Alzheimer’s Disease/Dementia/Parkinson’s Disease. Research demonstrates that hospice care improves end-of-life outcomes for adults with Alzheimer’s Disease and related dementias (ADRD), yet with eligibility limited to a six-month prognosis, hospice is not structured to meet longer-term needs. The result is a live discharge from hospice. In 2019, 17.4% of hospice patients were discharged alive from hospice, with 6.5% discharged due to being ‘no longer terminally ill.’ The majority of live discharges are either hospice-initiated due to patient stabilization (extended prognosis resulting in a situation in which a patient no longer meets the life expectancy hospice eligibility criteria) or are patient-initiated (revocation) where a patient or proxy chooses to leave hospice care, typically to access disease-directed therapies or inpatient hospitalization. Both present unique challenges and opportunities for hospice providers. Informed by over 10 years of practice experience and research, this presentation will discuss the impact of live discharge from hospice and the unique impact for patients and primary caregivers of individuals with ADRD, the service gaps that exist for this population, and our recommendations for policy reform.

